# Low physiologic oxygen tensions reduce proliferation and differentiation of human multipotent mesenchymal stromal cells

**DOI:** 10.1186/1471-2121-11-11

**Published:** 2010-01-28

**Authors:** Christina Holzwarth, Martin Vaegler, Friederike Gieseke, Stefan M Pfister, Rupert Handgretinger, Gunter Kerst, Ingo Müller

**Affiliations:** 1 University Children's Hospital, Department of General Pediatrics, Hematology and Oncology, Tübingen, Germany; 2 German Cancer Research Center and University Children's Hospital, Heidelberg, Germany; 3 University Children's Hospital, Department of Pediatric Cardiology, Pulmology and Intensive Care, Tübingen, Germany

## Abstract

**Background:**

Human multipotent mesenchymal stromal cells (MSC) can be isolated from various tissues including bone marrow. Here, MSC participate as bone lining cells in the formation of the hematopoietic stem cell niche. In this compartment, the oxygen tension is low and oxygen partial pressure is estimated to range from 1% to 7%. We analyzed the effect of low oxygen tensions on human MSC cultured with platelet-lysate supplemented media and assessed proliferation, morphology, chromosomal stability, immunophenotype and plasticity.

**Results:**

After transferring MSC from atmospheric oxygen levels of 21% to 1%, HIF-1α expression was induced, indicating efficient oxygen reduction. Simultaneously, MSC exhibited a significantly different morphology with shorter extensions and broader cell bodies. MSC did not proliferate as rapidly as under 21% oxygen and accumulated in G_1 _phase. The immunophenotype, however, was unaffected. Hypoxic stress as well as free oxygen radicals may affect chromosomal stability. However, no chromosomal abnormalities in human MSC under either culture condition were detected using high-resolution matrix-based comparative genomic hybridization. Reduced oxygen tension severely impaired adipogenic and osteogenic differentiation of human MSC. Elevation of oxygen from 1% to 3% restored osteogenic differentiation.

**Conclusion:**

Physiologic oxygen tension during *in vitro *culture of human MSC slows down cell cycle progression and differentiation. Under physiological conditions this may keep a proportion of MSC in a resting state. Further studies are needed to analyze these aspects of MSC in tissue regeneration.

## Background

Human multipotent mesenchymal stromal cells (MSC) obtained from bone marrow are characterized by a multilineage differentiation potential and a high proliferative capacity without losing their genetic stability [1]. By the mid of the last decade, the clinical potential was recognized [2]. Today, several clinical trials showed that the application of *ex vivo *expanded MSC is safe and feasible [3-5]. MSC have earned considerable attention as therapeutic tools in graft-versus-host disease after allogeneic hematopoietic stem cell transplantation [6]. The treatment of pediatric patients suffering from osteogenesis imperfecta was the first successful clinical application of MSC in regenerative medicine [3,7]. Another promising application of MSC has been explored in a pilot study where five children with steroid-induced osteonecrosis of the femur received MSC directly injected into the necrotic area of the bone [8]. Regenerative properties of MSC could also be demonstrated in the treatment of ischemic cardiovascular diseases [9].

These examples of tissue regeneration share the phenomenon of oxygen deprivation in the affected organs challenging the ability of MSC to differentiate into bone or other tissues [10]. Physiologically the cells are adapted to low oxygen levels with oxygen concentrations between 1% and 7%. Mathematical models of the pO_2 _distribution in human bone marrow suggest a gradient across the marrow from the relatively well oxygenated sinuses to the rather hypoxic endosteal region [11]. It is known that low oxygen tension is involved in keeping stem cells in a quiescent state retaining their plasticity [12]. Conversely, hypoxia may also serve as a danger signal and recruit MSC. In a rat model, Rochefort and colleagues showed that specifically MSC and not hematopoietic progenitor cells were mobilized out of the bone marrow into the peripheral blood by hypoxia [13]. However, it is an open issue if various oxygen concentrations over prolonged periods of time change the characteristic properties which define MSC *in vitro *as proposed by the International Society for Cellular Therapy [14]. Reports addressing this issue obtained conflicting results: In several *in vitro *studies, low oxygen concentrations have been found to be stimulating differentiation processes, exemplarily shown by inducing the cells toward the adipogenic, osteogenic or chondrogenic lineage [15-17]. In contrast, other groups reported suppressive effects of reduced oxygen tensions on MSC plasticity [10,18]. It is hypothesized that survival and proliferative capacity of MSC can be enhanced by maintaining the cells under low oxygen tensions. Standard culture of MSC at 21% O_2 _imply hyperoxic conditions compared to physiological O_2 _concentrations, i. e. 1% - 7%, with increased oxidative stress and subsequent chromosomal instability and thereby possibly contribute to the limited growth rates [19]. In addition, subtle differences of culture conditions such as growth factor supplementation of the media may contribute to the heterogeneity of the results.

MSC contribute to the niche for hematopoietic stem cells (HSC) in the bone marrow which consists of three compartments: the bony niches lined with osteoblasts proximal to the endosteal surface, the stromal niche contained within the sinusoidal endothelial cells in the medulla of the bone, and adipocytes [20]. It was shown in animal models that regional blood supply and level of oxygenation directly affect localization and stemness of HSC. The most primitive hematopoietic progenitor cells are sequestered in niches at the lowest end of an oxygen gradient where they are maintained in an undifferentiated state and mainly act via trophic factor secretion. Towards higher oxygen levels, expansion and differentiation of HSC increases [21,22].

We hypothesized that MSC and HSC share similar responses to varying oxygen concentrations concerning alterations in their phenotypic and functional properties. Therefore, we assessed morphology, proliferation kinetics, cell cycle characteristics, immunophenotype, plasticity and chromosomal stability of MSC maintained at atmospheric and physiological, i. e. low oxygen tensions. Regarding the discrepancies between previous studies in the field, it is important to note that platelet lysate was used as growth supplement, because it is gaining attention for clinical application of MSC. Moreover, prolonged incubation periods and minimal oxygen concentrations of 1% were used to mimic the microenvironment, which may also occur in therapeutic situations of regenerative medicine.

## Results

### Effect of physiological oxygen concentrations on MSC morphology

**Figure 1:**
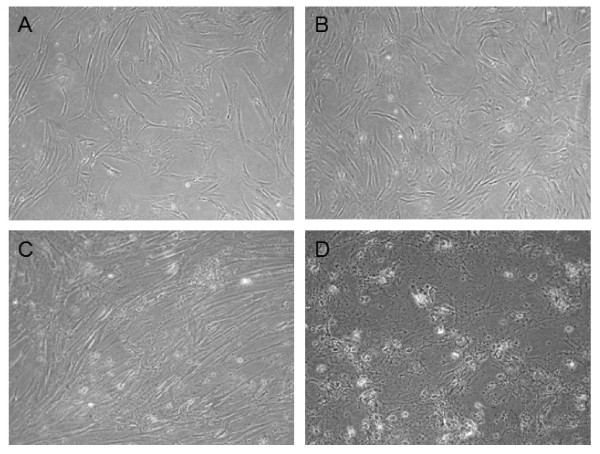
**Morphological evaluation of MSC under different oxygen conditions**. Photomicrographs of two representative MSC cultures from separate donors. In one culture no significant morphological difference after 3 weeks between 21% O_2 _(A) and 1% O_2 _(B) were found. In a second culture, MSC under 1% O_2 _did not adapt the typical triangular shape and became plastic-adherent, but died after 1 week (D), while the control cells maintained under 21% O_2 _(C) exhibited the characteristic MSC morphology. Obviously, donor-dependent factor greatly impact the growth and differentiation behavior of MSC under these conditions. Out of 10 cultures 3 showed growth under both oxygen tensions, whereas 7 displayed less tolerance to low oxygen tension of 1%.

The cell morphology under reduced oxygen tension and under atmospheric conditions was analyzed via light microscopy with photomicrographs taken at the time points of 1 week and 3 weeks. We observed two different behaviors and sensitivities to oxygen deprivation. Figure 1 shows photomicrographs of two representative donors: MSC of donor 1 cultured in either 21% O_2 _or 1% O_2 _over 3 weeks were morphologically indistinguishable. Both populations could be seen as colonies of triangular cells after several days of culture. As the cells adhered to plastic, the nonadherent hematopoietic cells in the culture were removed during changes of medium and virtually homogenous cultures with the characteristic confluent, spindle-shaped MSC were obtained after three passages. The population grown under atmospheric conditions of donor 2 showed the characteristic time course of MSC morphology as described for donor 1. In contrast, the population of donor 2 maintained in 1% O_2 _neither adapted the typical triangular shape nor did it become a confluent, plastic-adherent monolayer, but died after one week (D). Thus, MSC morphology under reduced oxygen tension is also donor dependent. These experiments were repeated with a total of ten different pediatric donors as summarized in table 1.

**Table 1:** Characteristics of MSC donors and MSC cultures.

Age in years	Sex	Status of MSC in 21% and 1% O_2_
1	M	Disintegration of MSC after 7 days under 1% O_2_

1	W	No differences after 3 weeks

4	M	No differences after 3 weeks

5	W	No MSC after 7 days under 1% O_2_

5	M	Disintegration of MSC after 7 days under 1% O_2_

6	M	No MSC after 7 days under 1% O_2_

11	M	No differences after 3 weeks

13	M	Disintegration of MSC after 7 days under 1% O_2_

14	M	No MSC after 7 days under 1% O_2_

18	M	Disintegration of MSC after 7 days under 1% O_2_

### Effect of reduced oxygen concentrations on MSC immunophenotype

**Figure 2:**
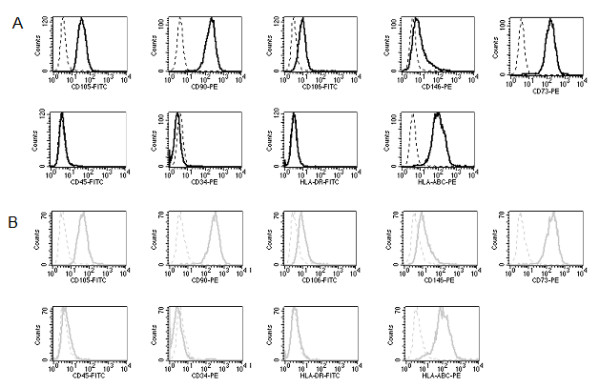
**Immunophenotyping profile of MSC under 21% O_2 _(A) and 1% O_2 _(B) by flow cytometric analysis of cell surface marker expression after 14 days showed no changes in immunophenotype of MSC due to varying oxygen concentrations (n = 3)**.

The immunophenotype of human MSC is characterized by the cell surface expression of CD73, CD90, CD105, CD106, CD146 and MHC class I. Furthermore, the absence of hematopoietic markers such as CD45 and of CD34 or MHC class II is used to identify culture expanded MSC. We analyzed these markers by flow cytometry in cells from three different donors cultured at 21% O_2 _and at 1% O_2_. Only cultures of donors whose cells survived at 1% O_2 _were used in this set of experiments. There was no significant difference in cell surface expression of the markers mentioned above after 14 days of culture at the indicated oxygen tension (figure 2).

### Effect of physiological oxygen concentrations on the expression of hypoxia-inducible factor-1α

**Figure 3:**
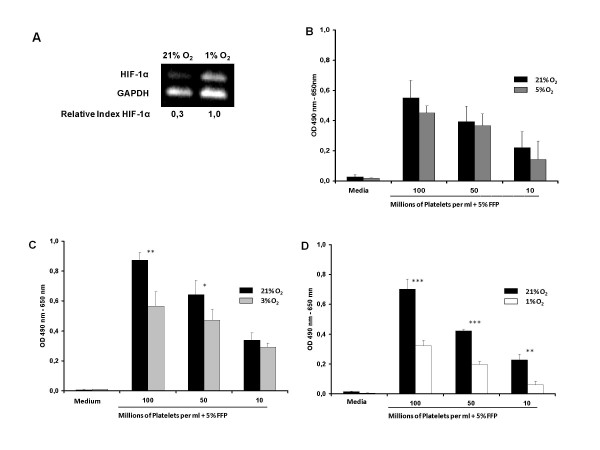
**HIF-1α upregulation in response to low oxygen tension**. Exposure of MSC to hypoxia resulted in a more than 3-fold increase of the HIF-1α expression as detected by semiquantitative RT-PCR. The relative index for HIF-1α was normalized against GAPDH relative intensity. Effect of reduced oxygen tension on MSC proliferation kinetics (A). Cell proliferation was measured by an increase in OD using the MTS assay after an incubation period of 7 days under 21% O_2_, 5% O_2 _(B), 3% O_2 _(C), and 1% O_2 _(D), respectively. Values indicate the mean and standard deviation for 3 cultures of each condition. Significance for all analysis was set at *** p < 0,001, ** p < 0,01, * p < 0,05 using student's *t*-test.

HIF-1α is induced at low oxygen concentrations. We investigated the mRNA levels of the transcription factor in MSC, which had been expanded at 21% O_2 _and 1% O_2 _in order to prove efficiency of oxygen reduction in our experimental setting. Under atmospheric culture conditions HIF-1α expression was detected at a low level as assessed by reverse transcription PCR. In contrast, the relative index for HIF-1α expression, which was obtained by normalizing HIF-1α against GAPDH intensity, increased more than 3-fold when the cells had been grown at low oxygen tensions. Hence, near physiological oxygen concentrations upregulated the expression of hypoxia-inducible factor-1α at the mRNA level (figure 3A).

### Effect of physiological oxygen concentrations on proliferation of MSC

The use of subatmospheric oxygen concentrations during *in vitro *culture of human MSC was found to be stimulating in some publications and inhibitory in others. To assess the effect of platelet-derived growth factors in our GMP-conform culture conditions, we analyzed proliferation of MSC under atmospheres of 21%, 5%, 3% and 1% O_2 _by an MTS proliferation assay. In our system, we found that hypoxia reduced MSC proliferation after an incubation period of seven days (figure 3B-D). At an oxygen concentration of 21%, which is hyperoxic in comparison to the physiological environment of MSC, cell proliferation was vigorous. The proliferation rate and metabolic activity declined depending on the oxygen concentration tested.

**Figure 4:**
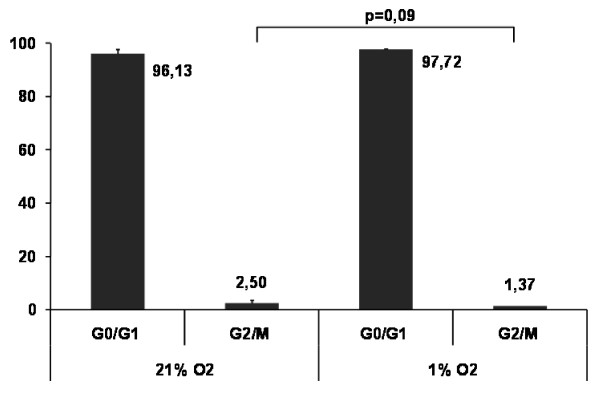
**Flow cytometric cell cycle analysis of MSC under atmospheric and reduced oxygen conditions**. Cells were permeabilized and stained with propidium iodide. DNA content-related cell cycle distribution of MSC after 7 days of incubation under 21% O_2 _and 1% O_2 _(n = 3).

To further corroborate these results, we analyzed cell cycle progression of MSC under 21% and 1% oxygen atmospheres. Figure 4 shows the cell cycle analysis of human MSC after a period of 7 days at the chosen culture conditions. Only 1.37% of MSC had entered the G_2_/M phase in the hypoxic cell culture in comparison to 2.50% at an oxygen concentration of 21%. Similar results were obtained after an adaptation period of 3 weeks (data not shown). This decrease of cells in the G_2_/M phase confirms the inhibitory effect of reduced oxygen concentrations on MSC proliferation in our experimental setting.

### Chromosomal stability of MSC at different oxygen tensions

**Figure 5:**
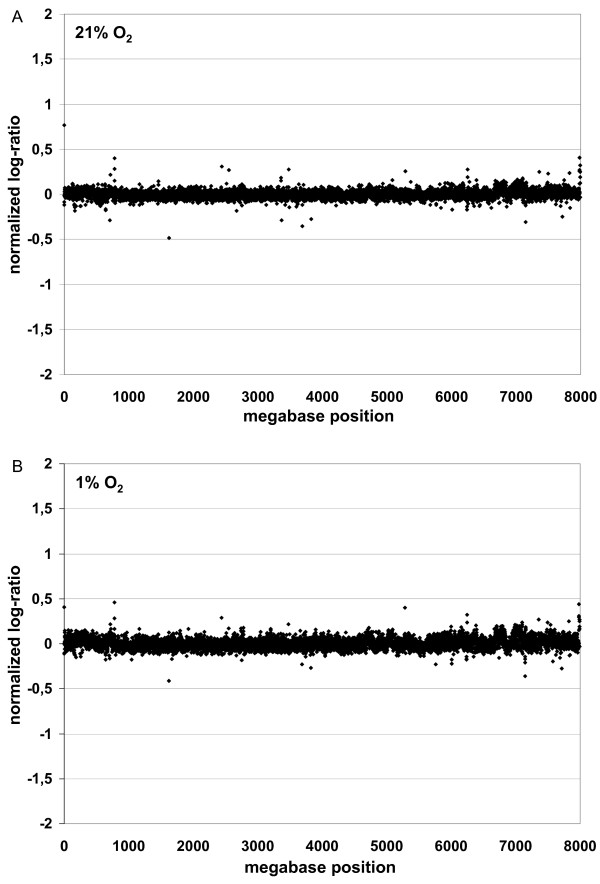
**Effect of hypoxia on chromosomal stability**. Chromosomal aberrations were not detectable by high-resolution matrix-based comparative genomic hybridization after 4 weeks of MSC culture at 21% O_2 _and 1% O_2_. Except for typical polymorphisms, the DNA of MSC did not show any chromosomal abnormalities compared to freshly isolated DNA from PBMC of healthy volunteers (n = 3).

Environmental stress by low and high oxygen concentrations, respectively, may affect chromosomal stability of MSC and thereby contribute to the different biological properties observed so far. Moreover, chromosomal stability of MSC under these conditions is relevant for preparation of MSC and clinical application in tissue regeneration. We performed high-resolution matrix-based comparative genomic hybridization of three independent MSC samples after 4 weeks of cell culture. No chromosomal aberrations were detected (figure 5).

### Effect of low oxygen concentrations on MSC plasticity

**Figure 6:**
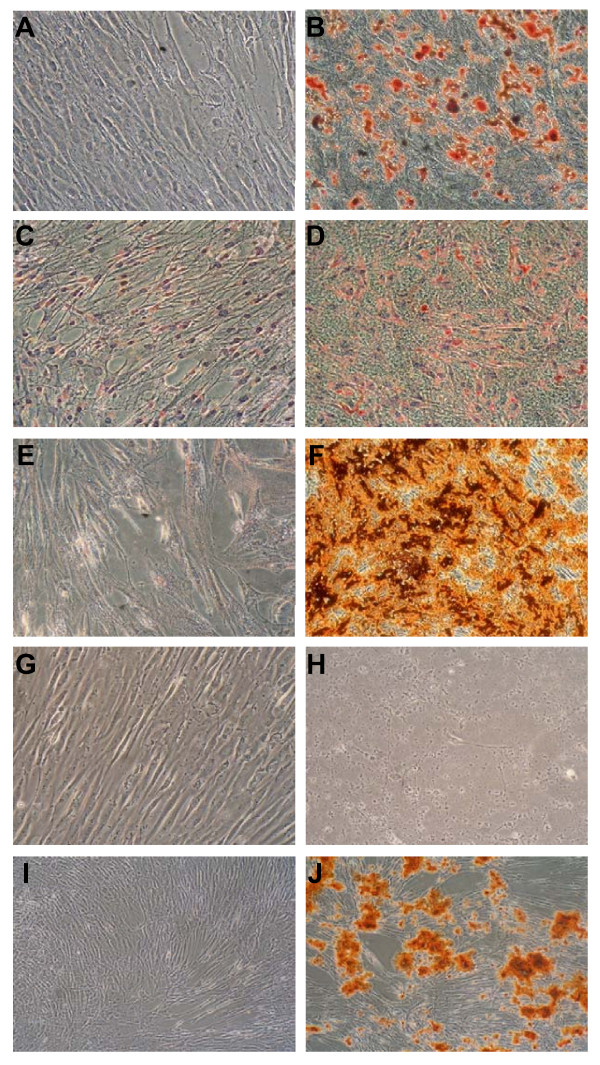
**Effect of hypoxia on MSC plasticity**. Oil-Red-O stain of MSC in adipogenic induction medium at atmospheric and reduced oxygen concentrations after 14 days of culture: (A) 21% O_2_. control; (B) 21% O_2_. adipogenic induction; (C) 1% O_2_. control; (D) 1% O_2_. adipogenic induction. Alizarin Red-S staining for the deposition of calcium precipitates is an indicator for the osteogenic differentiation. Viability and osteogenic differentiation of MSC from most donors was drastically reduced in this medium under 1% O_2 _as compared to 21% O_2_: (E) 21% O_2_. control; (F) 21% O_2_. osteogenic induction; (G) 1% O_2_. control; (H) 1% O_2_. osteogenic induction. However, a slight increase of oxygen to 3% allowed for efficient differentiation of MSC into osteoblasts: (I) 3% O_2_. control; (J) 3% O_2_. osteogenic induction. Three independent experiments of adipogenic and osteogenic differentiation were performed.

Besides growth factor secretion, the potential of MSC as therapeutic tools in regenerative medicine is determined by their ability to differentiate into various tissues. Therefore, MSC grown under reduced oxygen and atmospheric conditions were analyzed for their capability to differentiate into adipocytes and osteoblasts as proof of plasticity. Culture of MSC in adipogenic induction medium for 14 days under 21% O_2 _resulted in the appearance of adipocyte-like cells containing lipid droplets, which stained positive with Oil-Red-O (figure 6B). The same phenomenon could be observed with cells cultured at 1% O_2_, but to a lesser extent (figure 6D). The osteogenic differentiation potential under both conditions was evaluated by Alizarin Red-S staining of calcium precipitates after 14 days of culture in osteogenic induction medium. Figure 6F shows that MSC differentiated into osteoblasts at 21% O_2 _as the deposition of calcium precipitates could be detected, but not at 1% O_2 _(figure 6H). To demonstrate how sensitive the system is towards small changes in oxygen tension, we performed the osteogenic differentiation at 3% O_2 _as well. Interestingly, these assays showed no detectable difference in comparison to 21% O_2 _(figure 6J).

## Discussion

Oxygen concentration during standard *in vitro *culture of primary human cells is often not adapted to the *in vivo *situation. The bone marrow is a compartment with an oxygen concentration of 1% to 7%, increasing from the endosteal lining cells to the sinusoid lining cells [11]. It is becoming evident that oxygen is a regulator of stem cell biology [23]. As MSC in bone marrow are usually found as bone lining cells, they are exposed to considerably lower oxygen concentrations *in vivo *than *in vitro*. This artificial situation has prompted several groups to investigate key features of MSC with conflicting results [10,12,15,16,18,19]. The inconsistency of these results was possibly due to a number of variations in the experimental setup, including (i) use of MSC from different species, namely rodents and humans; (ii) different media composition, in particular source and amount of growth factors; and (iii) subtle differences in the oxygen concentrations used from 8% to 1%. This prompted us to investigate human MSC, which are kept in media supplemented with platelet lysate and human plasma [24-28]. This media is increasingly used for expansion of human MSC prior to clinical applications under GMP-conform conditions [8,29].

We found that human MSC in platelet-lysate supplemented media show a reduced growth rate under oxygen supply close to what is expected *in vivo*. This finding is in agreement with data obtained for hematopoietic stem cells [21] as for MSC [10]. In the stem cell niche, which is located close to the endosteum, low oxygen supply keeps a stem cell pool in a slowly cycling state and maintains plasticity. Here, the role of HIF1α, which is upregulated under hypoxic conditions, plays an important role in concert with bFGF [30]. Furthermore, HIF1α sensitizes cells to bFGF and vice versa [31,32]. These oberservations are candidate mechanisms for maintaining MSC in a more quiescent, more plastic state, because bFGF was shown to maintain plasticity in a closely related fibroblastic population isolated from the nucleus pulposus [33]. Differences in bFGF-content in FCS and human plasma/platelet lysate may contribute to some of the different results obtained in previous reports.

MSC in a slowly cycling state may be more protected from DNA damage caused by errors during replication as well as from free oxygen radical species. In this respect it is not surprising that MSC do not proliferate and differentiate under reduced oxygen levels as compared to atmospheric oxygen levels. Although we have not detected chromosomal aberration during the culture period in our CGH assay, mutations could be favored by high oxygen tensions during conventional culture conditions. We therefore limit the culture period in our institution for clinical application of human MSC to four weeks as a safety measure.

These results may contribute to a better understanding of the regenerative potential displayed by MSC in ischemic tissue. Several groups including our own have shown, that MSC do not only contribute to tissue repair by transdifferentiation, but also by secretion of trophic factors or angiogenic cytokines [8,34,35]. It may depend to a large extent on other cytokines in the damaged tissue and even on the donor, how MSC stimulate tissue regeneration in a given setting. The microenvironment will play an important role in the biological function of MSC. Even minimal changes have significant impact on biological properties of MSC as can be seen from the fact that increasing the oxygen from 1% to 3% during osteogenic induction almost completely restored this plasticity (figure 6). The situation of regenerating necrotic tissue cannot be compared with the stem cell niche in bone marrow. Inflammatory cytokines are present in the areas of ischemic tissue in high concentrations [36]. Therefore, the biological behavior of MSC is most likely different in both situations, as MSC are sensitive to proinflammatory cytokines and change their gene expression profile rapidly [37,38].

## Conclusions

Our data demonstrate that human MSC show reduced proliferation rates and accumulation in G_1 _phase when cultured in platelet lysate supplemented media under strongly reduced oxygen concentrations. Moreover, MSC exhibit a pronounced reduction in differentiation into adipose and bone tissue under these conditions as compared with atmospheric oxygen. Future experiments directed at gene expression profiling under these conditions in the presence or absence of proinflammatory cytokines may help to understand the mechanisms underlying the quiescent state and the regenerative activity of human MSC.

## Methods

### Isolation and culture of MSC

MSC were obtained from excessive material of diagnostic bone marrow aspirates of children with hematopoietic malignancies after informed consent and approval by the local IRB (241/2005V). MSC cultures were established as described earlier [25]. Briefly, bone marrow samples (0.5 ml) were heparinized, subjected to red blood cell lysis using the ammonium chloride method, and washed with Hank's buffered salt solution (Lonza, Basel, Switzerland). Cells were placed in low glucose Dulbecco's Modified Eagle Medium (LG-DMEM, Lonza) supplemented with 5% (v/v) human fresh frozen plasma (FFP), 10^7^/ml platelets (University of Tübingen blood donor centre), 80 IU/ml heparin sulphate, 1 mM glutamine (Lonza), 100 IU/ml penicillin, 100 μg/ml streptomycin (both Biochrom, Berlin, Germany) in one well of a six-well culture plate. Nonadherent cells were purged on day 2 and medium was replaced twice a week. At 80% confluence, cells were harvested with trypsin 0.5% (Lonza) and replated in the above medium at 2,000 cells/cm^2^. To expand the cells, successive passages were performed with the same protocol for a maximum of six weeks.

Equal numbers of culture dishes of the same donor were kept in Heracell gas addition incubators (Heraeus Instruments GmbH, Hanau, Germany) with gas mixtures consisting of either 21% O_2_, 74% N_2_, 5% CO_2 _(referred to as 21% O_2_), 1% O_2_, 94% N_2_, 5% CO_2 _(referred to as 1% O_2_) or 3% O_2_, 92% N_2_, 5% CO_2 _(referred to as 3% O_2_) for at least one week before performing an experiment.

### Proliferation

After 7 days of adaptation in 21%, 3% and 1% O_2_, respectively, MSC were plated in a 96 well plate at a density of 6,250 cells/cm^2 ^and cultured for another 7 days. Proliferation was analyzed using the MTS assay kit according to the instructions of manufacturer (Promega, Madison, WI).

### Cell Cycle Analysis

Cell cycle analysis of MSC was performed after 7 days and 21 days of adaptation in 21% O_2 _and 1% O_2_. 10^6 ^MSC were washed with phosphate buffered saline (PBS, Biochrom, Berlin, Germany) three times, fixed with ice-cold 70% ethanol and stored at 4°C for a minimum of 1 hour. After three more washing steps with PBS, DNA and RNA of the MSC were stained with propidium iodide/RNase staining buffer (BD Biosciences, Heidelberg, Germany). The samples were incubated at room temperature for 15 minutes and analyzed by flow cytometry on a FACS Calibur (Becton Dickinson, Heidelberg, Germany).

### Differentiation assays

The differentiation of MSC was assessed after an adaptation period of 14 days in 21% O_2 _and 1% O_2 _as reported earlier [25].

**Adipogenic differentiation **was induced in 80% confluent MSC cultures by supplementing the cell culture medium with 1 μM dexamethasone, 60 μM indomethacin, 0.5 mM isobuthylmethylxanthine (all Sigma-Aldrich, Steinheim, Germany) and 10 μM insulin (Novo, Novodisk, Bagsværd, Denmark). After 14 to 20 days, histochemical staining with Oil-Red-O (Sigma) was performed to detect lipid droplet formation.

**Osteogenic differentiation **was induced in 50% confluent MSC cultured in cell culture medium supplemented with 10 nM dexamethasone and 0.1 mM L-ascorbic acid-2-phosphate (both Sigma) from day 0 to day 7. For 7 to 14 more days, 10 mM beta-glycerol phosphate (Sigma) and 100 ng/ml bone morphogenic protein-2 (Tebu-Bio, Magenta, Italy) were added as additional cell culture supplements. To verify the osteogenic differentiation, calcium precipitates were detected by aqueous 0.5% (v/v) Alizarin Red-S (Sigma) histochemical staining. This differentiation was also performed at 3% O_2_.

### Immunophenotyping

MSC of the same donor were placed in 21% O_2_, 3% O_2 _or in 1% O_2 _atmospheres for 14 days. Subsequently, MSC were harvested, washed with PBS supplemented with 2% Fetal Calf Serum (FCS, Biochrom) and stained with the following antibodies: anti-IgG1-FITC (cloneMOPC-31C), anti-IgG1-PE (clone G18-145), anti-CD45-FITC (clone HI30), anti-CD34-PE (clone 563), anti-CD73-PE (clone A02), anti-HLA-DR-FITC (clone TÜ36), anti-HLA-ABC-PE (clone G46-2.6), all from Becton Dickinson and anti-CD105-FITC (clone N1-3A1, Ancell, Bayport, MN, USA), anti-CD90-PE (clone F15-42-1), anti-CD106-FITC (clone 1.G11B1, both Serotec, Düsseldorf, Germany) and anti-CD146-PE (clone P1H12 from Santa Cruz Biotechnology, Heidelberg, Germany). The immunophenotype was analyzed using a FACS Calibur and CellQuest analysis software (Becton Dickinson).

### Array-based Comparative Genomic Hybridization (array-CGH, matrix-CGH)

Array- (or Matrix-) CGH [39] was carried out as previously described [40-42]. Selection of genomic clones, isolation of BAC DNA, performance of DOP-PCR, preparation of microarrays, labeling, hybridization and washing procedures were performed as outlined. Raw data processing and normalization was performed as previously reported providing log_2_-ratios of spot intensities [43]. The chromosomal mapping information is based on the University of California at Santa Cruz (UCSC) genome database (March 2006) and the March 2006 (hg18) assembly (NCBI Build 36.1) of the International Human Genome Sequencing Consortium. All clones from totally 11 samples were subjected to pre-processing, which included elimination of clones with incomplete mapping information or missing data in more than 20% of cases. This resulted in 7980 clones evaluable for further analysis. In the next step, genomic events were assigned. The median of all MADs (median absolute deviation) across all chromosomes is taken to estimate the sample experimental variability (genomic "standard deviation", SD_g_). Gains and losses are defined by log_2_-ratios larger than 3 times SD_g _or smaller than - 3 times SD_g_, respectively.

### Semiquantitative Reverse Transcriptase-Polymerase Chain Reaction

After 4 weeks of expansion in 21% and 1% O_2_, MSC were harvested and total RNA was extracted using peqGOLD TriFast™ reagent (PEQLAB Biotechnologie GmbH, Erlangen) according to the manufacturer's instructions. Complementary DNA (cDNA) was synthesized from 1 μg total RNA (Invitrogen, Groningen, The Netherlands) and amplification was done using the following primers: HIF-1α, for: CTCAAAGTCGGACAGCCTCA/rev: CCCTGCAGTAGGTTTCTGCT; GAPDH, for: CGGGAAGCTTGTGATCAATGG/rev: GGCAGTGATGGCATGGACTG. PCR conditions were: HIF-1α, 94°C for 3 min/95°C for 30 s, 56°C for 30 s, 72°C for 1 min (20 cycles)/72°C for 10 min; GAPDH, 95°C for 2 min/95°C for 30 s, 55°C for 30 s, 72°C for 30 s (20 cycles)/72°C for 7 min. Products (358 bp) were separated on 2% agarose gels, visualized with ethidium bromide and analyzed using the gel documentation system AIDA 1D Evaluation (Raytest Isotopenmessgeräte GmbH, Straubenhardt, Germany).

## Authors' contributions

CM performed cell culture experiments and data analysis; MV performed experiments and data analysis; FG performed cell culture experiments and data analysis; SMP performed CGH array and data analysis; RH participated in study design; GK participated in the study design and drafted the manuscript; IM participated in the study design and drafted the manuscript. All authors read and approved the final version of manuscript.
